# Single-cell immune aging clocks reveal inter-individual heterogeneity during infection and vaccination

**DOI:** 10.1038/s43587-025-00819-z

**Published:** 2025-03-05

**Authors:** Wenchao Li, Zhenhua Zhang, Saumya Kumar, Javier Botey-Bataller, Martijn Zoodsma, Ali Ehsani, Qiuyao Zhan, Ahmed Alaswad, Liang Zhou, Inge Grondman, Valerie Koeken, Jian Yang, Gang Wang, Sonja Volland, Tania O. Crişan, Leo A. B. Joosten, Thomas Illig, Cheng-Jian Xu, Mihai G. Netea, Yang Li

**Affiliations:** 1 Department of Computational Biology of Individualised Medicine, Centre for Individualised Infection Medicine (CiiM), a Joint Venture Between the Hannover Medical School and the Helmholtz Centre for Infection Research, Hannover, Germany; 2 TWINCORE, Centre for Experimental and Clinical Infection Research, a Joint Venture Between the Hannover Medical School and the Helmholtz Centre for Infection Research, Hannover, Germany; 3 Department of Internal Medicine and Radboud Center for Infectious Diseases, Radboud University Medical Center, Nijmegen, The Netherlands; 4 Research Centre Innovations in Care, Rotterdam University of Applied Sciences, Rotterdam, The Netherlands; 5 The National Clinical Research Center for Mental Disorders and Beijing Key Laboratory of Mental Disorders, Beijing Anding Hospital, Capital Medical University, Beijing, China; 6 Hannover Unified Biobank, Hannover Medical School, Hannover, Germany; 7 Department of Medical Genetics, Iuliu Haţieganu University of Medicine and Pharmacy, Cluj-Napoca, Romania; 8 Cluster of Excellence RESIST (EXC 2155), Hanover Medical School, Hannover, Germany; 9 Lower Saxony center for artificial intelligence and causal methods in medicine (CAIMed), Hannover, Germany; 10 Department for Immunology and Metabolism, Life and Medical Sciences Institute (LIMES), University of Bonn, Bonn, Germany

**Keywords:** Computational biology and bioinformatics, Immunology, Ageing

## Abstract

Aging affects human immune system functionality, increasing susceptibility to immune-mediated diseases. While gene expression programs accurately reflect immune function, their relationship with biological immune aging and health status remains unclear. Here we developed robust, cell-type-specific aging clocks (sc-ImmuAging) for the myeloid and lymphoid immune cell populations in circulation within peripheral blood mononuclear cells, using single-cell RNA-sequencing data from 1,081 healthy individuals aged from 18 to 97 years. Application of sc-ImmuAging to transcriptome data of patients with COVID-19 revealed notable age acceleration in monocytes, which decreased during recovery. Furthermore, inter-individual variations in immune aging induced by vaccination were identified, with individuals exhibiting elevated baseline interferon response genes showing age rejuvenation in CD8^+^ T cells after BCG vaccination. sc-ImmuAging provides a powerful tool for decoding immune aging dynamics, offering insights into age-related immune alterations and potential interventions to promote healthy aging.

## Abstract (web summary)

Immune system function changes substantially with age. In this study, the authors present single-cell transcriptomic aging clocks revealing immune-cell-specific age acceleration that is shaped by infection and vaccination, as well as identifying pathways and regulators of immune system aging.

## Main

Aging substantially impacts the human immune system, with two main functional consequences: a decrease in immune cell responsiveness, combined with increased inappropriate systemic inflammation (also called “inflammaging”). This leads to both a reduced ability to defend against external or internal challenges^1^ and increased susceptibility to inflammation-driven disorders such as atherosclerosis and neurodegenerative diseases^2,3^. While chronological age is a major indicator of biological aging, the substantial heterogeneity in the kinetics of immune aging among individuals of the same chronological age suggests that chronological time alone does not fully account for the changes observed^4^. Hence, measuring “biological age” rather than merely lifespan can more accurately and comprehensively assess the process of aging^5^, offering a unique view of age-related immune biology.

Although studies during the past decade have identified a relatively large number of age-related biomarkers, the intricate nature of biological aging presents challenges to fully uncover its underlying mechanisms. The concept of an “aging clock” emerged as a promising approach by using machine-learning methods to comprehensively capture the dynamics of aging through the integration of aging-related markers at the molecular level.

As a hallmark of aging^6^, DNA methylation-based epigenetics data were the first to be used to establish epigenetic aging clocks^7–10^, which have been widely used to predict both lifespan and healthspan. The impact of specific diseases on epigenetic aging clocks has been also reported, such as age acceleration in coronavirus disease 2019 (COVID-19)-infected individuals^11^ and hepatitis C virus-infected individuals^12^. In the field of immune biological aging, ref. ^13^ proposed an inflammatory aging clock (iAge) based on proteomics data and identified that the chemokine CXCL9 is an important player in age-related chronic inflammation. The immune aging score (IMM-AGE), developed by the group of Shen-Orr and colleagues, is an immune system aging clock that uses longitudinal data on inflammatory markers to estimate an individual’s immune status, showing its ability to predict age-related health outcomes and mortality risks^14^. It is worth noting that age-related changes are also evident at the level of gene expression. One of the first transcriptomic aging clocks, proposed in 2015, used age-related marker genes identified from a meta-analysis of whole blood RNA sequencing data^15^. Subsequently, ref. ^16^ developed a machine learning-based aging clock for human fibroblasts derived from skin tissues but lacked independent datasets to evaluate the generalizability and robustness of the model. There is growing evidence that aging impacts various immune cells in a heterogeneous manner^17^, and single-cell RNA-sequencing (scRNA-seq) technologies provide the potential to develop cell-type-specific aging clocks. While the first single-cell transcriptome aging clock was proposed in 2023, focusing on mouse brain tissue^18^, research into aging in the human immune system at the single-cell resolution remained unexplored.

Given the important role of circulating immune cells in immune responses, examining their functional changes attributable to age largely improves our understanding of susceptibility to infections, cancer, and inflammatory diseases. As vaccination efficacy experiences a substantial decline with advanced age^19^, such knowledge would also be important to improve vaccination strategies. In this Article, we established a robust cell-type-specific aging clock, sc-ImmuAging, covering monocytes, CD4^+^ T cells, CD8^+^ T cells, natural killer (NK) cells, and B cells, based on single-cell transcriptomic profiles of 1,081 samples of peripheral blood mononuclear cells (PBMCs) collected from European healthy adults. Both internal and external validations consistently showed the outperformance of our constructed models. We also demonstrated the value of these aging clocks to identify notable age alterations in monocytes after COVID-19 infection and to reveal the heterogeneity of age alterations in CD8^+^ T cells among individuals vaccinated with BCG (Bacillus Calmette–Guérin). Our research sheds light on understanding biological age alterations in response to vaccinations and diseases, indicating its potential application in improving personalized disease treatment and assessing age rejuvenation intervention.

## Results

### Single-cell transcriptome aging clocks predict biological age

**Fig. 1:**
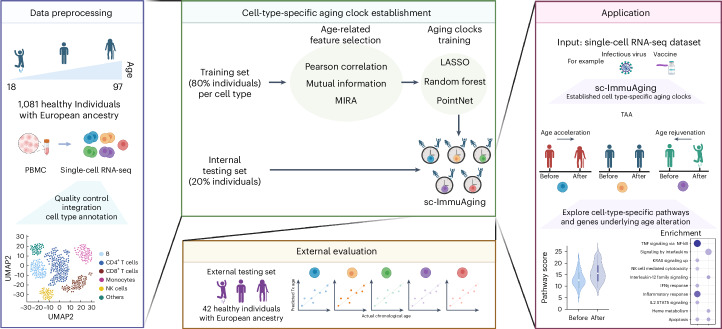
Workflow of establishing cell-type-specific aging clocks, sc-ImmuAging. The establishment and application of our aging clocks involve four main steps. (1) Data pre-processing: five publicly available scRNA-seq datasets from human PBMC are collected, including 1,081 healthy European individuals aged from 18 to 97 years. Next, quality control, integration, and cell-type annotation are conducted. In the following analysis, we focus on five major cell types, including B cells, NK cells, CD4^+^ T cells, CD8^+^ T cells and monocytes. (2) Model establishment: 80% of individuals are randomly selected for model training. Pearson correlation, mutual information and MIRA are used and compared in terms of feature selection. The selected features are trained by LASSO, random forest, and PointNet, respectively. Finally, cell-type-specific aging clocks are developed for each cell type. Subsequently, evaluation is performed using an internal testing set, including 20% individuals. See Methods for details. (3) External validation: An independent external validation dataset of 42 healthy European individuals from five published datasets is used. Then the Pearson correlation coefficient, r.m.s.e., and m.a.e. between actual age and predicted transcriptome age (Tx age) are calculated to evaluate the accuracy of the model. (4) Application: We apply our established cell-type-specific aging clock model on vaccination and infectious diseases cohorts as case studies. We aim to examine which cell types exhibit the substantial age acceleration/rejuvenation in response to infectious diseases or vaccination. Furthermore, we explore the genes and pathways that contribute to age alterations. Fig. 1 was created with BioRender.com.

**Extended Data Fig. 1:**
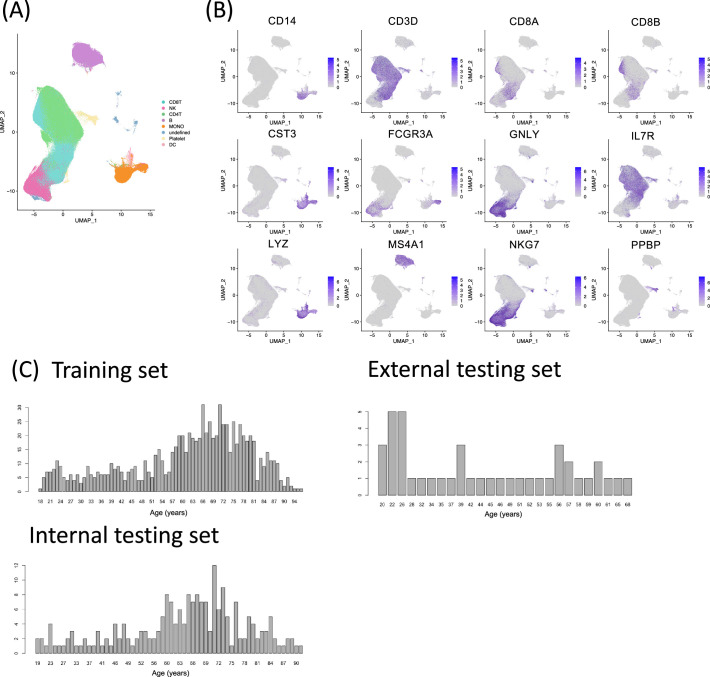
Overview of collected scRNA-seq datasets. (**a**) UMAP of the scRNA-seq data. (**b**) Expression of marker genes for cell type annotations. (**c**) Age distributions for training set, internal testing set, and external testing set, respectively.

We developed immune cell-type-specific transcriptome aging clocks, sc-ImmuAging, for human PBMCs (Fig. 1), using scRNA-seq datasets from five studies^20–24^, encompassing 1,081 healthy individuals of European ancestry aged 18 to 97 years (Methods). After quality control, approximately 1.3 million high-quality cells were retained for further analysis (Methods) (Extended Data Fig. 1a,b). Focusing on the five most prevalent immune cell types in the circulation within PBMCs, CD4^+^ T cells, CD8^+^ T cells, monocytes, NK cells, and B cells, we built independent aging clocks for each using machine learning (least absolute shrinkage and selection operator (LASSO)^25^ and random forest^26^) and deep learning (PointNet^27^) methods, assessing performance through various metrics.

**Fig. 2:**
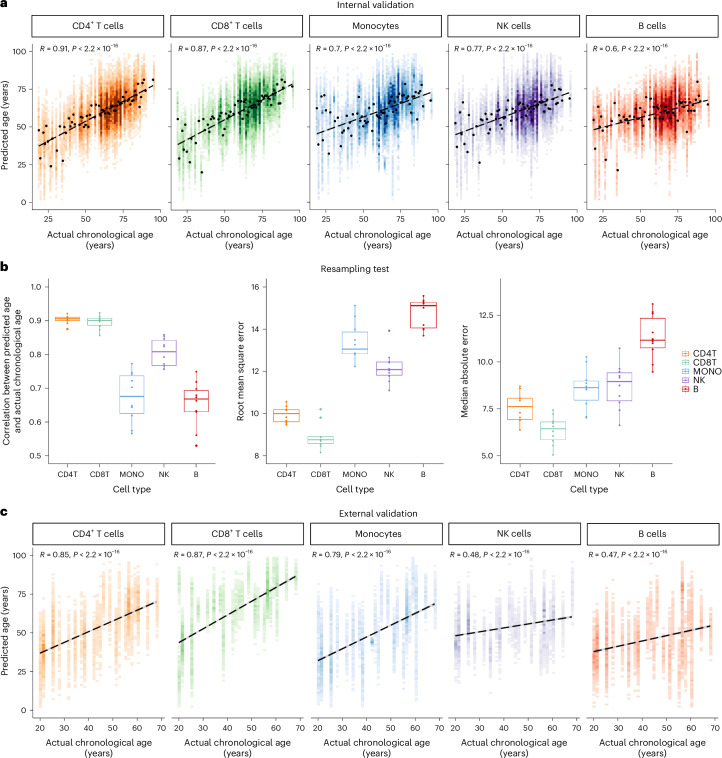
Evaluations on internal and external dataset show accuracy and robustness of aging clocks. **a**, Internal validation: Pearson correlation (two-sided test) is plotted between actual chronological age and predicted age for each cell type. Linear fit is plotted as a dashed line. **b**, To verify the robustness of the aging clock models, the training set and internal validation set are randomly resampled, and the model is trained for 10 times. Pearson correlation (two-sided test), r.m.s.e., and m.a.e. are used as evaluation criteria. In the boxplots: center, median; box limits, upper and lower quartiles; points, resampling; whiskers, 1.5× interquartile range. CD4T, CD4^+^ T cells; CD8T, CD8^+^ T cells; MONO, monocytes; NK, NK cells; B, B cells. **c**, External validation: Pearson correlation (two-sided test) is plotted between actual chronological age and predicted age in each cell type. Linear fit is plotted as a dashed line. Source data

We evaluated aging clocks performance by comparing the predicted and chronological ages (Pearson’s *R*, root mean square error (r.m.s.e.), and median absolute error (m.a.e.)). A cross-validation step was implemented in which 80% (864 individuals) were randomly selected for model training, while the remaining 20% (217 individuals) constituted the internal validation set. A comprehensive evaluation of different methods used for building aging clocks is summarized in Extended Data Table 1. LASSO regression showed superior performance across cell types. The strong correlation between chronological age and predicted transcriptomic age (*R* = [0.6, 0.91]; Fig. 2a and Extended Data Table 2) underscored the accuracy of our aging clocks. Through a 10-times repeated cross-validation analysis, we ensured the robustness of our aging clock models, with subtle performance variations observed across iterations, in terms of Pearson’s *R*, m.a.e., and r.m.s.e. (Fig. 2b). While PointNet, a deep learning approach, exhibited comparable performance in constructing cell-type-specific aging clocks (Extended Data Table 3), we proceed with the LASSO regression model due to its interpretability advantages.

**Extended Data Fig. 2:**
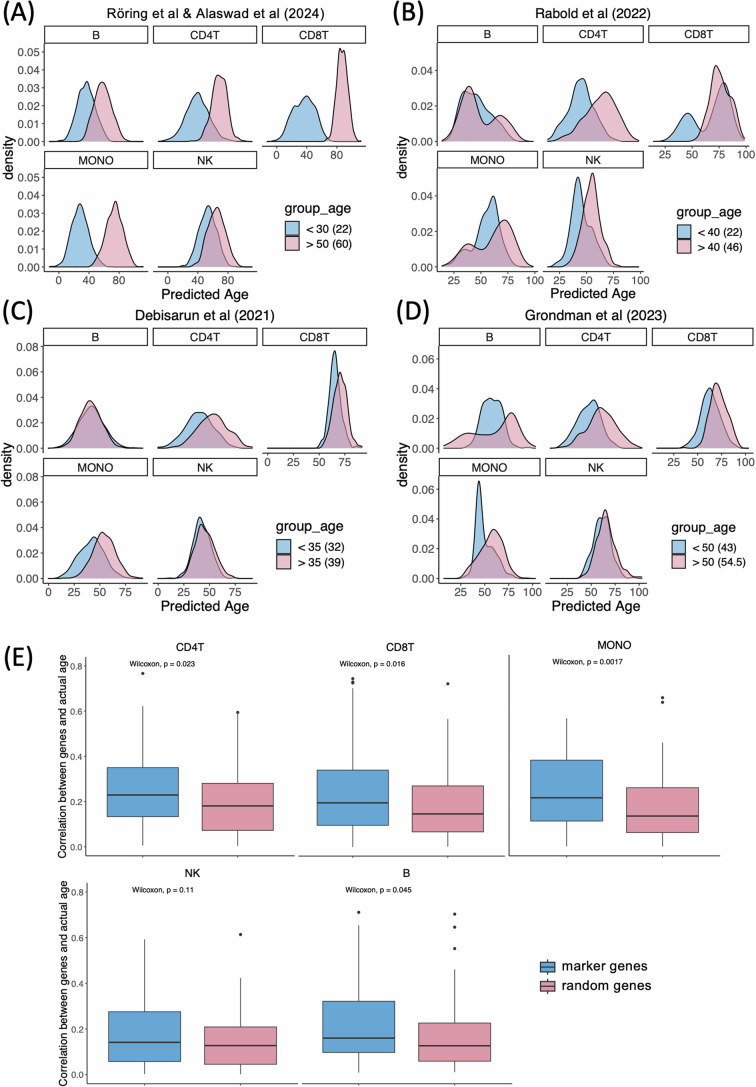
External validation performance. (**a**) External validation on Röring et al.^30^ dataset, with average chronological age of 22 years, and Alaswad et al.^32^ dataset, with average chronological age of 60 years. Density plots of predicted age for these two datasets are compared. (**b**) Individuals from Rabold et al.^28^ dataset were categorized into those under 40 years old (average actual chronological age = 22, blue density plot) and over 40 years old (average actual chronological age = 46, red density plot), based on age distribution of the samples. Density plots represent the predicted age for each group. (**c**) Individuals from Debisarun et al.^29^ dataset are split into those under 35 years old (average actual chronological age = 32, blue density plot) and above 35 years old (average actual chronological age = 39, red density plot). Density plots represent the predicted age for each group. (**d**) Individuals from Grondman et al.^31^ dataset are grouped into those under 50 years old (average actual chronological age = 43, blue density plot) and over 50 years (average actual chronological age = 54.5, red density plot). Density plots represent the predicted age for each group. (**e**) Boxplot of comparison between top 100 marker genes (ranked by absolute coefficient) of each aging clock and randomly selected genes using training set (n = 864). The y axis is the −log_10_(*P* value) (two-sided wilcoxon test). Blue color means genes captured by the model and red color means random genes. boxplots: centre, median; box limits, upper and lower quartiles; whiskers, 1.5× interquartile range. Source data

To assess the generalization ability of sc-ImmuAging, we tested it on independent datasets consisting of an additional 42 healthy individuals collected from five studies (healthy individuals from a thyroid cancer cohort, a sepsis cohort, an influenza cohort, a MMR (measles, mumps, and rubella vaccine) cohort, and a gout cohort)^28–32^. Similar age distribution of both training and external validation sets is shown in Extended Data Fig. 1c. The results showed reliable predictions, confirming the efficacy of the transcriptional aging clocks (Fig. 2c and Extended Data Fig. 2a–e).

In summary, our developed single-cell transcriptome aging clocks, sc-ImmuAging, accurately predict immune aging across various ages and cell types, offering avenues for exploring both the biology of aging and the dynamics of immune aging alteration during infection and vaccination at a single-cell resolution.

### Characterizing marker genes for cell-type aging clocks

For each of the cell-type-specific aging clocks constructed above, hundreds of marker genes whose expression levels correlate with age were selected as predictors of biological age (Supplementary Table 1). These aging marker genes provide insights into the molecular mechanisms underlying aging and age-related diseases.

**Fig. 3:**
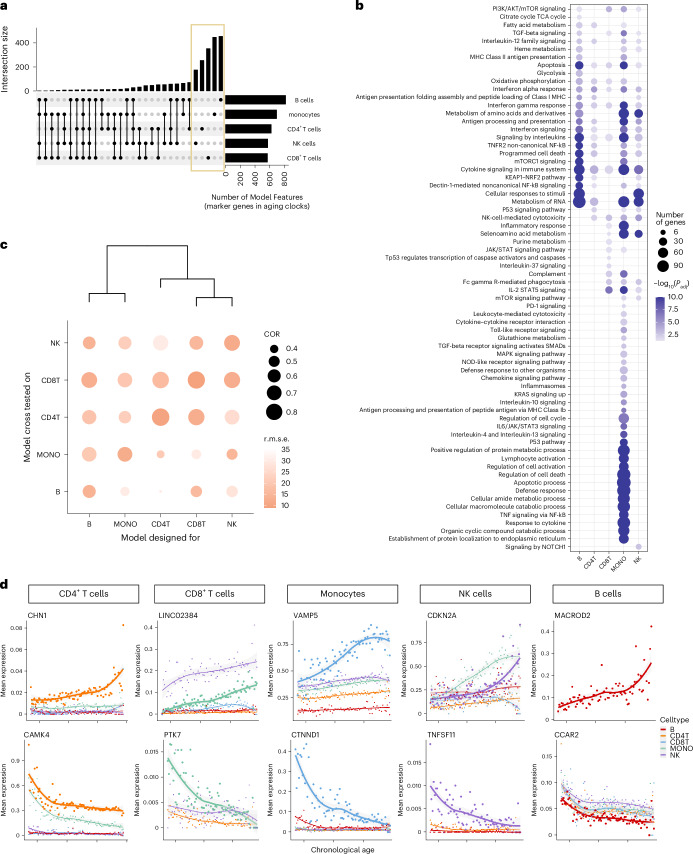
Characteristics of the established cell-type-specific aging clocks. **a**, The upset plot of the intersections of the captured marker genes, with non-zero coefficients, between cell-type-specific aging clocks. **b**, Top enriched pathways of the captured marker genes in each cell-type-specific aging clock model. Pathways with Benjamini–Hochberg adjusted *P* < 0.05 are considered significant. **c**, Evaluation of the specificity of cell-type-specific aging clocks across cell types. The *x* axis shows the cross tested of each model across cell types, and the *y* axis represents the trained model. Dot size denotes the Pearson correlation (COR) between actual chronological age and predicted age. The darkness of color represents the r.m.s.e. **d**, Trajectory of mean expression of top marker gene in each cell type. The genes on the first row have positive coefficients, and genes in the second row have negative coefficients. Source data

Directly comparing selected marker genes across cell types revealed a considerable proportion unique to each cell type (Fig. 3a), while enriched pathways could still be shared (Fig. 3b). Pathway enrichment analysis using these marker genes revealed common pathways such as interferon-gamma (IFNγ) response, apoptosis, and cytokine signaling across all cell types, consistent with previous research linking inflammation to aging^33^. In addition, pathways related to interleukin-12 and cytotoxicity were enriched in multiple cell types, aligning with existing literatures^34–36^.

As the marker genes of different cell types’ aging clocks share similar functions, we aimed to assess the performance of these aging clocks for predicting immune aging across various cell types. As illustrated in Fig. 3c, we observed the highest correlation and lowest r.m.s.e. between predicted and chronological age for the corresponding cell types. However, when applying an aging clock designed for one cell type to predict the age of another cell type, the performance was less accurate, indicating the specificity of the constructed aging clocks (Fig. 3c). Nevertheless, aging clocks showed similar predictability across similar immune cell types, such as CD4^+^ T cells and CD8^+^ T cells, which are consistent with previous study^37^. We further applied a bulk aging clock^15^ to the pseudo-bulk data generated from our single-cell dataset and found that it performed less effectively compared to our single-cell aging clocks. These analyses underscore the importance of cell-type-specific aging clocks.

To validate our marker genes, we compared them with reported aging-related genes from human whole blood^15^. Among the shared marker genes, a substantial proportion exhibited the same direction across cell types, notably in CD4^+^ T cells (75%) and CD8^+^ T cells (62%) (Extended Data Table 4). Examining the gene expression trajectories of identified marker genes with age further supported our findings, revealing the expected age-dependent expression patterns for markers in their corresponding cell types (Fig. 3d). For instance, the marker gene *CAMK4* in CD4^+^ T cells, known for its role in immune response and inflammation^38^, exhibited expression dynamics consistent with previous report^15^. Similarly, *MACROD2*, identified as the top marker gene for the B-cell aging clock and exhibiting an increased aging-dependent expression specifically in B cells, is recognized as a prominent feature in age-associated B cells^39^. These results underscore the importance of establishing cell-type-specific aging clocks and validate the accuracy of our selected marker genes.

### Monocytes show aging shifts in response to COVID-19 infection

**Fig. 4:**
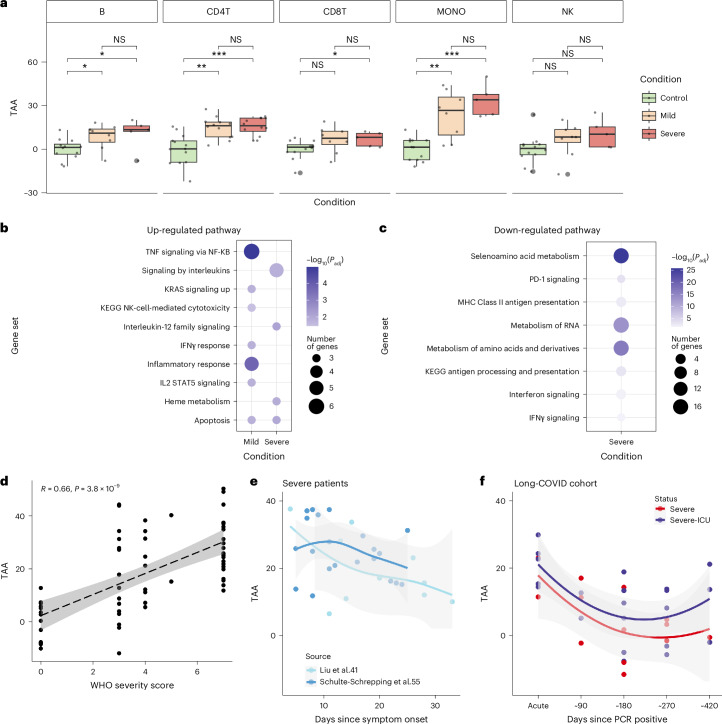
Monocytes undergo substantial age acceleration in response to COVID-19. **a**, Boxplot of the age shifts in healthy control and patients with mild and severe COVID-19 in each cell type, calculated as the predicted age subtract by the actual chronological age. See details in Methods. Boxplots: center, median; box limits, upper and lower quartiles; whiskers, 1.5× interquartile range. Two-sided Wilcoxon test: *P*_B:control-mild_ = 0.0314, *P*_B:control-severe_ = 0.0485, *P*_CD4T:control-mild_ = 0.0011, *P*_CD4T:control-severe_ = 0.000449, *P*_CD8T:control-severe_ = 0.0348, *P*_MONO:control-mild_ = 0.00298, *P*_MONO:control-severe_ = 0.000323; NS, not significant. **b**, Enriched pathways are calculated by the intersected genes between marker genes and up-regulated genes in patients with mild/severe COVID-19 compared to healthy control in monocytes. Pathways with Benjamini–Hochberg adjusted *P* < 0.05 are considered significant. **c**, Enriched pathways are calculated by the intersected genes between marker genes and down-regulated genes in patients with mild/severe COVID-19 compared to healthy control in monocytes. Pathways with Benjamini–Hochberg adjusted *P* < 0.05 are considered significant. **d**, Correlation between age shifts in monocytes and WHO severity score. Dashed line represents the linearly fitted curve between WHO severity score and TAA, with the 95% confidence interval (CI). **e**, Time series age shifts of patients with severe COVID-19 from two independent published scRNA-seq datasets. The *x* axis is days since symptom onset, and the *y* axis is the age shifts. Curve is fitted using “loess” function, with the 95% CI. **f**, Time series age shifts of severe and severe ICU patients from a single-nucleus RNA-seq dataset. ICU, intensive care unit. The *x* axis is days since positive PCR test result, and the *y* axis is the age shifts. Curve is fitted using loess function, with the 95% CI. Source data

**Extended Data Fig. 3:**
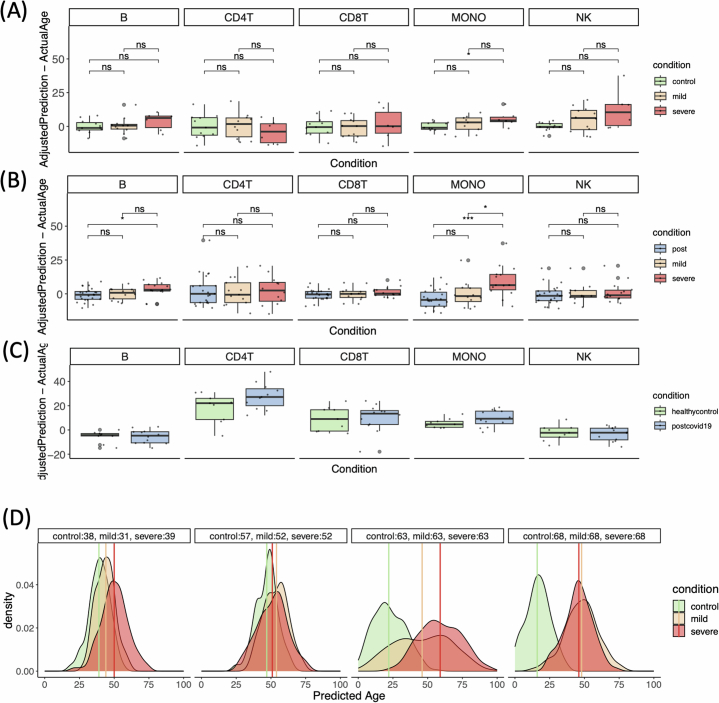
Application of sc-ImmuAging on the independent COVID-19 cohorts. (**a**) Boxplot of TAA for the Stephenson et al.^22^ cohort (n = 26). boxplots: centre, median; box limits, upper and lower quartiles; whiskers, 1.5× interquartile range. *two-sided wilcoxon test:* *P*_*MONO*:*control*-*severe*_ = 0.0202; ns, not significant. (**b**) Boxplot of TAA for Zhang et al.^56^ cohort (n = 48). boxplots: centre, median; box limits, upper and lower quartiles; whiskers, 1.5× interquartile range. *two-sided wilcoxon test:* *P*_*MONO*:*post*-*severe*_ = 0.000336, *P*_*MONO*:*mild*-*severe*_ = 0.0107; ns, not significant. (**c**) Boxplot of TAA for Liu et al.^24^ cohort (n = 24). boxplots: centre, median; box limits, upper and lower quartiles; whiskers, 1.5× interquartile range. (**d**) Density plot of the predicted age for healthy control, mild and severe patients. The median value of each distribution is highlighted as a vertical line. Four groups of individuals with different age stages are plotted. Source data

We next applied sc-ImmuAging to investigate the immune aging alterations during and after infection at a single-cell level. Using single-cell transcriptome data from four independent cohorts of patients with COVID-19, we first estimated the transcriptome age alteration (TAA) defined as the predicted age difference between patient and control at the same age (Methods). Our analysis revealed a notable transcriptome age acceleration (TAA > 0) caused by infection across immune cell types, especially in patients with severe COVID-19 (Fig. 4a). It is worth noting that monocytes showed the strongest and most consistent age acceleration across all the cohorts (Extended Data Fig. 3a–c). Given these findings, we focused on monocytes and compared TAA for age-matched individuals of control, mild, and severe conditions. Age acceleration was observed in monocytes from all patients with COVID-19, with a higher acceleration noted in patients with severe COVID-19 compared to those with mild COVID-19 across various age groups (Extended Data Fig. 3d).

To investigate the biological pathways underlying age acceleration due to severe acute respiratory syndrome coronavirus 2 (SARS-CoV-2) infection, we performed enrichment analysis of the genes that were both marker genes and differentially expressed in patients compared to control (Fig. 4b,c). We found up-regulation of TNF signaling via nuclear factor-κB (NF-kB) in patients with mild COVID-19, consistent with validated activation post infection^40,41^. Patients with severe COVID-19 showed elevated circulating concentrations of pro-inflammatory cytokines^42^, which are paradoxically often accompanied by poor responsiveness to secondary stimulation, aligning with the observed up-regulation of interleukin signaling pathways. In addition, up-regulated genes in both patients with mild and patients with severe COVID-19 were all enriched in apoptosis pathways, suggesting a potential contribution of these up-regulations to aging acceleration. The down-regulated IFN signaling pathway in patients with severe COVID-19 corroborated previous reports of suppressed Type I IFN production in severe cases^43,44^.

Furthermore, a Pearson’s correlation analysis revealed a significant positive association between WHO (World Health Organization) severity score and TAA (*P* = 6.66 × 10^−6^; Fig. 4d). Applying sc-ImmuAging to two independent time series COVID-19 cohorts revealed a decrease in age acceleration in monocytes during the days after the symptoms started to subside (Fig. 4e). This pattern was further replicated in an independent single-nucleus dataset from a long-COVID cohort, where TAA was highest during the acute phase and decreased over time. The majority of samples exhibited age acceleration even after 90 days from the onset of infection, possibly due to the impact of long COVID on the immune system, as these patients all exhibited dysfunctional lung capacity (Fig. 4f).

In summary, our sc-ImmuAging applied to patients with COVID-19 revealed notable aging acceleration in monocytes, with further analysis identifying relevant pathways involved in COVID-19 pathogenesis. The observed decrease in age alterations over time suggested a gradual return of biological ages to healthy states.

### Inter-individual variations in vaccination-induced immune age

**Fig. 5:**
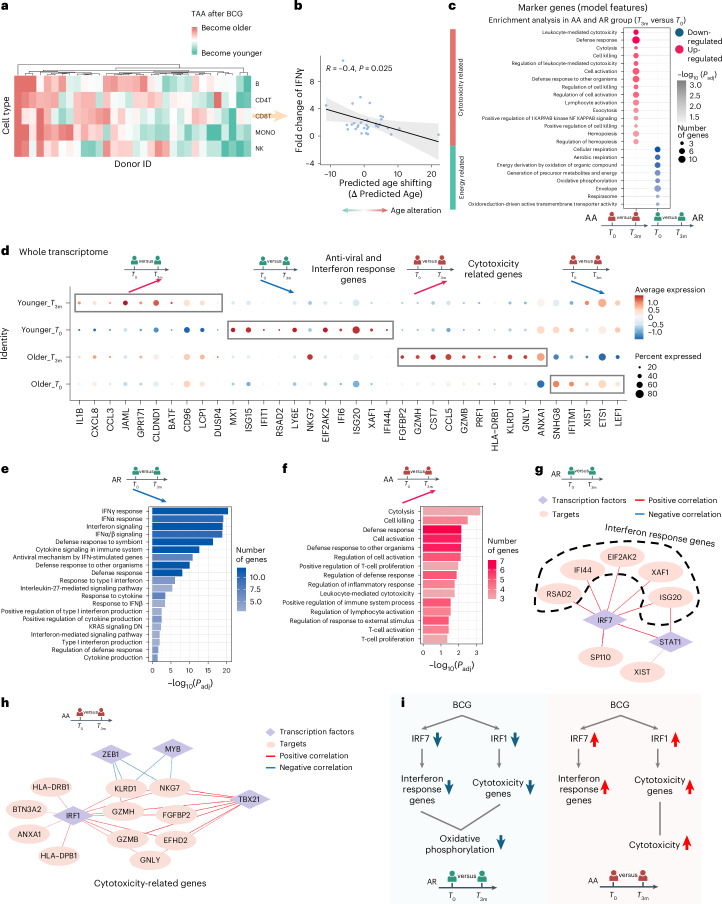
CD8^+^ T cells exhibit diverse age alterations among individuals in response to BCG vaccination. **a**, Age shifts in each cell type per individual. Red color means age acceleration after BCG vaccination, and green means the opposite. **b**, Significant correlation (Pearson correlation, *P* = 0.025, *R* = 0.4) between BCG efficacy, defined as the fold change of IFNγ between before and after vaccination with *M. tuberculosis* stimulation, and age shifts in CD8^+^ T cells. We define individuals with age rejuvenation as AR group and individuals with age acceleration as AA group. **c**, Enrichment analysis by using the genes which are marker genes and DEGs (compared between before (*T*_0_) and after (*T*_3m_) BCG vaccination, *P* < 0.05) in AR and AA groups. **d**, Dot plot of DEGs (adjusted *P* < 0.05), comparing before and after BCG vaccination at the whole transcriptome level in AR and AA groups, respectively. **e**, Pathway enrichment analysis of down-regulated genes after BCG vaccination compared with before BCG vaccination in the AR group. Pathways with Benjamini–Hochberg adjusted *P* < 0.05 are considered significant. **f**, Pathway enrichment analysis of up-regulated genes after BCG vaccination compared to before BCG vaccination in the AA group. Pathways with Benjamini–Hochberg adjusted *P* < 0.05 are considered significant. **g**, Unique gene regulatory network in the AR group. Targets are down-regulated after BCG vaccination. Diamonds refer to transcription factors, and circles refer to targets. The color of edges represents correlation between transcription factors and targets. Red means positive correlation, and blue means negative correlation. **h**, Unique gene regulatory network in the AA group. Targets are up-regulated after BCG vaccination. Diamonds refer to transcription factors, and circles refer to targets. The color of edges represents correlation between transcription factors and targets. Red means positive correlation, and blue means negative correlation. **i**, Schematic plot summarizing the age shifts and the involved biological processes in CD8^+^ T cells in AR group and AA group, respectively. Panels **c**–**i** were created using BioRender.com. Source data

Next, we investigated aging clock dynamics using sc-ImmuAging in immune cells from individuals before and 3 months after BCG vaccination. Our analysis revealed substantial inter-individual variations in BCG-induced TAAs across various immune cells (Fig. 5a), with individuals exhibiting either accelerated (AA) or rejuvenated (AR) TAA. It is worth noting that we found a significant negative correlation (Pearson correlation coefficient = −0.4, *P* = 0.025; Fig. 5b) between BCG-induced TAAs in CD8^+^ T cells and vaccine efficacy. This efficacy was measured by the fold change in IFNγ production upon *Mycobacterium tuberculosis* stimulation between before and after BCG vaccination, a well-established marker of BCG efficacy^45,46^. These findings are consistent with previous research indicating the active involvement of CD8^+^ T cells in BCG-induced biological processes^47^.

To investigate the biological pathways underlying BCG-induced changes in transcriptome age, we performed an enrichment analysis of genes that both served as marker genes of the transcriptomic aging clock and were differentially expressed in CD8^+^ T cells before and after vaccination (Fig. 5c). Our analysis revealed that energy-related pathways, such as oxidative phosphorylation, were down-regulated following vaccination in the group decreased TAA. This finding is consistent with previous research indicating a decrease in oxidative phosphorylation after BCG administration, suggesting a reset in cellular metabolism^48,49^. Conversely, cytotoxicity-related pathways were up-regulated in the AA group showing increased TAA, which may suggest a contribution of these pathways to the relatively poorer vaccine responses observed in this group (Fig. 5c).

To gain a deeper understanding of the differences between the AR and AA groups after BCG vaccination, we systematically analyzed the expression dynamics within CD8^+^ T cells from each group. Our examination revealed that individuals in the AR group exhibited higher expression levels of anti-viral and IFN response genes before vaccination compared to those in the AA group (Fig. 5d). Following BCG vaccination, these genes were substantially down-regulated in the AR group, likely contributing to the observed rejuvenation in transcriptome age after vaccination. Conversely, individuals in the AA group exhibited a notable up-regulation of cytotoxicity-related genes following BCG vaccination, a pattern not observed in the AR group in which these genes remained lowly expressed. This observed pattern was further confirmed through enrichment analysis based on the differential expressed genes (Fig. 5e,f).

To further validate the higher level of cytotoxicity in the AA group, we tested whether the fold change in the proportion of CD8^+^ terminally differentiated effector memory T cells re-expressing CD45RA (TEMRA) correlates with TAA. We found a significant positive correlation (*P* = 0.0074), indicating that individuals showing age acceleration have an increased proportion of CD8^+^ TEMRA cells after BCG vaccination. This finding is consistent with the elevated expression level of cytotoxicity pathways in the CD8^+^ T cells of the AA group.

Our subsequent goal was to identify the transcription factors that potentially regulate vaccination-induced changes in TAA. Through a transcription factor enrichment analysis^50^, we uncovered distinctive associations between transcription factors and IFN response genes in the AR group, highlighting the pivotal role of *IRF7* (Fig. 5g). Many of the IFN pathway genes undergoing decreased expression after BCG vaccination were under control of *IRF7*, potentially contributing to the observed age rejuvenation. By contrast, within the AA group, we observed an up-regulation of *IRF7* and its predicted targets after BCG vaccination. Further examination of the gene regulatory network in the AA group revealed *IRF1* as the primary transcription factor governing cytotoxicity genes (Fig. 5h). It is worth noting that previous research has underscored the significance of IRF1 in inducing T-cell cytotoxicity^51^. Also, we observed a down-regulation of *IRF1* and its target genes in the AR group after vaccination.

As summarized in Fig. 5i, following BCG vaccination, individuals can be stratified into AA and AR groups based on the calculated age alteration (that is, TAA) in CD8^+^ T cells using our aging clocks. In the AR group, BCG administration led to a decrease in the expression level of *IRF7*-dependent IFN response genes, while at the same time *IRF1*-dependent cytotoxicity genes were down-regulated. Conversely, the pattern observed in the AA group showed opposite directions of change, with high *IRF1*-dependent cytotoxicity genes. These findings shed light on the intricate regulatory mechanisms underlying age alterations in response to BCG administration, with *IRF7* and *IRF1* playing distinct roles in modulating IFN response and cytotoxicity, respectively, and potentially contributing to the differential outcomes observed in the TAA after vaccination.

**Extended Data Fig. 4:**
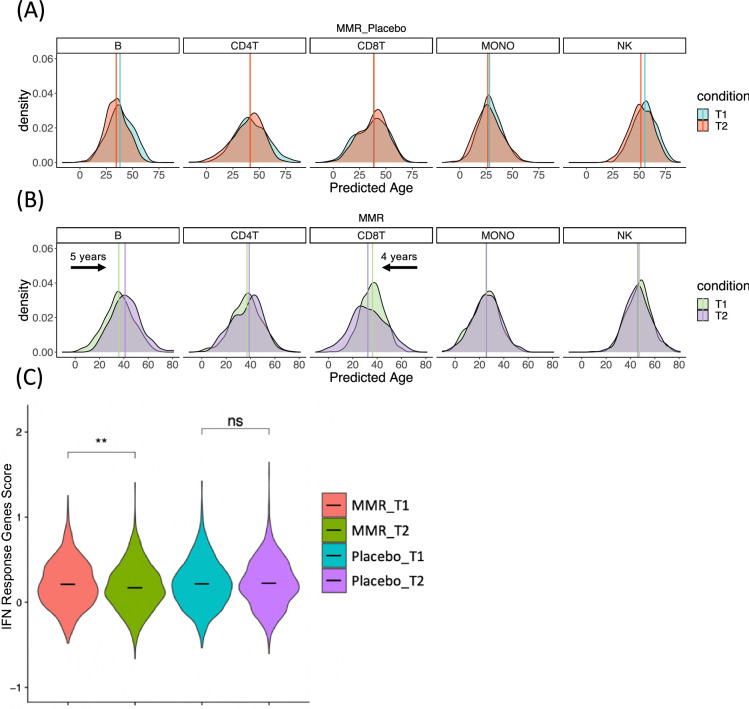
Application of sc-ImmuAging on the MMR cohort^30^. (**a**) Density Plot of the predicted age of the Placebo-vaccinated group at before and after administration. Vertical line represents the median predicted age of each group. No differences are detected between two timepoints, suggesting the prediction accuracy of our model. (**b**) Density Plot of the predicted age of the MMR-vaccinated group at before and after administration. Vertical line represents the median predicted age of each group. We observe five years age acceleration in B cells, whereas four years age rejuvenation in CD8 + T cells. (**c**) Violin plot of the Interferon response gene score (Methods) for the MMR- and Placebo-vaccinated group at two time points. The y axis is the interferon gene score. We observe a notable decrease in the MMR-vaccinated group after administration, which is consistent with the result from the BCG-vaccinated group. No significant changes are observed in the Placebo-vaccinated group. *two-sided wilcoxon test: P*_*MMR*:*T*1-*T*2_ = 0.0019; ns, not significant. Source data

To validate these findings, we conducted a similar analysis using scRNA-seq data from nine individuals both before and 4 weeks after receiving either the MMR vaccine or a placebo (Methods). In the placebo-vaccinated group, we did not observe any notable age alterations, as expected and confirming the validity of sc-ImmuAging (Extended Data Fig. 4a). When comparing predicted ages of CD8^+^ T cells before and after MMR vaccination, we found that 3 out of 4 individuals exhibited lower TAA, equivalent with immune age rejuvenation (Extended Data Fig. 4b). It is worth noting that we observed a substantial decrease in the expression levels of IFN response gene after MMR vaccination (Extended Data Fig. 4c and Methods), which mirrors the findings observed in the BCG-induced AR group.

## Discussion

In this study we collected scRNA-seq data from PBMCs of 1,081 healthy European individuals aged from 18 to 97 years and developed a cell-type-specific transcriptomic aging clock, sc-ImmuAging, to investigate the immune aging alterations in response to infection and vaccination. Through comprehensive internal and independent external validation, our proposed transcriptomics aging clocks exhibited an accurate and robust performance, enabling its potential for further application to gain a fundamental understanding of the impact of various diseases and vaccinations effect on the biological aging of the immune system. The marker genes captured by our models are cell type specific. For instance, age-related changes in *TNFSF11* were found to be specific to NK cells. It has been hypothesized that this gene may play a role in the regulatory process of apoptosis^52^. *PTK7* exhibited a unique age-dependent trajectory in CD8^+^ T cells: this gene has been proven to be associated with apoptosis in human bone marrow immune progenitors^53^. As these marker genes presented diverse trajectories with age across different immune cell types, this suggests a model of cell-type-specific transcriptional aging in immune cells. In addition, sex may markedly influence the accuracy of aging clocks, as reported by ref. ^54^. However, our results (not shown) indicate that overall prediction accuracy does not notably benefit from separate sex-specific analysis, likely due to reduced power from the smaller sample sizes for each sex-based subset. Future studies with larger collections of single-cell RNA-seq data for aging clock construction will allow us to refine and enhance the accuracy of sex-specific aging clocks.

To validate the functional implication of sc-ImmuAging, we extended our analysis to predict the biological age of individuals subjected to vaccination or suffering from an infection. In individuals with COVID-19, monocytes showed the most striking changes compared to other cell types, aligning with previous studies positing monocytes as key immunological players in the pathophysiology of SARS-CoV-2 infection^55,56^. By assessing two independent cohorts, the age acceleration in monocytes, which was strongly induced in the acute phase of the infection, consistently decreased within days since symptoms appeared. This observation was independently validated when we applied our aging clock of monocytes to single-nucleus data including patients with long COVID, with the accelerated biological ages gradually returning toward normal levels. While these data show the increase of immune aging during the acute phase, with a gradual return to normal levels during recovery, these findings raise an important question: does the post-infectious TAA decrease reach the normal immune age as before infection, or does a “residual TAA” persist after recovery? Even if such a residual TAA may be relatively minor (for example, 5% of the increased TAA during the acute phase), this would have big consequences in time if an individual is prone to recurrent infections. Such a scenario is supported by a study in Bangladeshi children in a highly infectious environment, showing accelerated immune aging compared with American counterparts^57^. Our sc-ImmunAging score provides an important tool to assess this hypothesis in future studies, as well as the possibility to assess the efficacy of potential interventions to slow immune aging.

Vaccinations are the most effective approach to decrease susceptibility to infections. By mimicking an infection, they induce long-term immune effects that are on the one hand protective but on the other hand could also impact immune aging. We therefore aimed to investigate such potential effects and as a model of vaccination. We explored the age alterations in response to BCG administration, one of the most used vaccines worldwide. It is also important to observe that BCG-induced transcriptional age alteration shows a notable inter-individual variation. It is worth noting that the changes in transcriptome age alteration (TAA) induced by BCG vaccination in CD8^+^ T cells negatively correlated IFNγ production upon ex vivo *M. tuberculosis* stimulation (*P* = 0.025). Using mycobacterial growth inhibition assays to evaluate *M. tuberculosis* growth control, we found no substantial correlation between phenotype from these assays and TAA before and after BCG vaccination. We used the Horvath aging clock^58^ to calculate epigenetic age alteration before and after BCG vaccination. However, we did not find a notable correlation between epigenetic age alteration and BCG efficacy. This result highlights the unique insights provided by single-cell transcriptome aging clocks. We further categorized individuals who responded to BCG vaccination with either a decreased (AR) or increased (AA) TAA, and we explore the differences between these groups at the molecular level. One first important observation was a decrease in transcription of genes important for oxidative phosphorylation in the BCG-vaccinated group showing decreased TAA: this observation argues for a role of oxidative metabolism in immune aging, and it is in line with the anti-aging properties of metformin—an inhibitor of mitochondrial complex I^59^. Considering the distinct transcriptome landscape between young and old individuals, in our analysis, only young individuals were considered to reduce potential biases. Future studies could include the individuals with a wider age range to evaluate the effects of vaccination on immune aging across different age groups.

To gain more insights in the molecular mechanisms responsible for changes in immune aging after vaccination, we performed a comparison of the whole transcriptome between AA and AR groups. We subsequently revealed that IFN response and cytotoxicity pathways were enriched among down-regulated genes in the AR group after BCG. Combining public databases with our scRNA-seq data, *IRF7* and *IRF1* were identified to regulate IFN response genes and cytotoxicity genes, respectively. By contrast, in individuals that experienced an increased TAA after BCG vaccination, the IFN genes under *IRF7* regulation were increased. This observation was further independently validated in a group of individuals vaccinated with MMR, suggesting that vaccines likely have consistent effects on immune aging and *IRF7*/*IRF1*-regulated pathways are likely to regulate these effects. An important area of future investigation will be to understand the factors that determine the heterogeneity of *IRF7*/*IRF1* pathways and TAA effects in different individuals after vaccination. Collectively, it was demonstrated that sc-ImmuAging can be applied in diverse PBMC datasets, with consistent results verifying the reliability of this model and the potential implementation in understanding human immune system in response to vaccine and infectious diseases.

While our prediction model exhibited an accurate prediction (*R* = 0.91) in CD4^+^ T cells, it is crucial to acknowledge certain limitations and the need for further improvement. First, the performance on external validation datasets in NK cells and B cells need to be improved by future studies. The reasons behind the less robust performance in B cells and NK cells remain unclear, and one potential approach to address it is including additional datasets for validation and exploration. To ensure the robustness and accuracy of sc-ImmuAging, we concentrated our efforts on the five main cell types with sufficient cell numbers from PBMCs. However, immune cell subpopulations exhibit distinct transcriptional profiles that play crucial roles in aging^60,61^. Therefore, further exploration of rare immune cell populations, such as dendritic cells and γδT cells, holds great promise. Complementary technologies such as SMART-seq3^62^ provide high-resolution single-cell data, offering a valuable opportunity for future validation of single-cell transcriptome aging models. In addition, neutrophils are recognized for their involvement in inflammation^63^ and their capacity to acquire long-term memory^64^. Investigating the immune age alterations of neutrophils would be highly valuable in future studies. Another limitation is that our training, testing, and application focused exclusively on the European population from whom enough datasets were available. It is our intention to pursue the assessment across populations of African and Asian ancestry in future work. Another area of great interest in the future is to intersect transcriptomic aging clocks and the known epigenetic aging clocks. The integration between these two layers of analysis or even other omics data could yield even deeper insights into aging alteration^65^. Beyond chronological age, current studies are moving forward to include clinical information as features or train the model based on age-related phenotypes. For instance, the second-generation methylation aging clock is more closely associated with mortality risk due to the inclusion of additional clinical parameters as features^66^ or because it is trained on aging-related phenotypes, such as telomere length^67^.

Looking ahead, our sc-ImmuAging holds promising potential for applications in personalized medicine. It can be used to evaluate the efficacy of drugs in slowing down or even reversing aging in individuals. It is worth noting that sc-ImmuAging has already shown its ability to capture inter-individual heterogeneity of age shift in response to vaccination, revealing that some individuals exhibit signs of rejuvenation while others may age more rapidly. This capability can be further explored, enabling the tailoring of interventions designed to promote rejuvenation. In addition, sc-ImmuAging can help determine the most appropriate vaccinations or drugs to administer based on the individual’s unique molecular profile. For instance, recent studies^68^ have indicated that metformin can decelerate the aging clock in male monkeys, showcasing a potential avenue for therapeutic application. Furthermore, as illustrated in our COVID-19 case study, sc-ImmuAging serves as a powerful tool for identifying molecular markers within specific cell types that drive aging in the context of disease. These insights could ultimately pave the way for targeted therapeutic strategies aimed at improving age-related conditions.

Overall, our study proposed cell-type-specific transcriptomic aging clocks, sc-ImmuAging, using scRNA-seq dataset with considerable sample size from human PBMCs, with the capacity to characterize the key molecules involved in immune responses and aging, thus contributing substantially to aging studies in human immunity. In addition to the biological insights of this study, sc-ImmunAging provides a framework to investigate immune aging in various diseases and monitor future approaches aimed to slow biological aging.

## Methods

### Study cohorts

#### OneK1K cohort

The Data1 dataset^21^ was accessed from CELLxGENE (https://cellxgene.cziscience.com/collections/dde06e0f-ab3b-46be-96a2-a8082383c4a1), which is a cohort including 981 individuals, aged above 17 years at recruitment. Healthy individuals without any stimulations were included in our study for model training and internal validation. PBMCs were barcoded with the Single Cell 3′ Library and Gel Bead Kit (10x Genomics) and sequenced by Illumina NovaSeq 2000. CellRanger (v2.2.0) was used to generate a gene expression matrix. The processed RDS file was downloaded, and raw counts were extracted from the Seurat object.

#### COVID-19-UK cohort

The Data2 dataset^22^ was accessed from European Molecular Biology Laboratory's European Bioinformatics Institute (EMBL-EBI) (https://www.ebi.ac.uk/biostudies/arrayexpress/studies/E-MTAB-10026), which is a cohort including 143 individuals from London, Newcastle, and Cambridge. Only PBMCs from 11 healthy individuals aged above 17 years without any stimulations were included into model training. Included in the case study were 26 patients with mild and 15 patients with severe COVID-19. In detail, the Newcastle cohort used Chromium NextGEM Single Cell V(D)J Reagent kits v1.1. Cambridge used Single Cell V(D)J 5′ version 1.1 (1000020), while London used 10 × 5′ single-cell capture (Chromium Next GEM Single Cell V(D)J Reagent Kit v1.1). All three cohorts were sequenced and processed according to the 10x Genomics manufacturer’s protocols. PBMcs were sequenced by Illumina NovaSeq 6000. CellRanger (v4.0) was used to generate a gene expression matrix. The processed h5ad file was downloaded, and raw counts were extracted from the Seurat object.

#### Influenza A virus (IAV) cohort

The Data3 dataset^23^ was accessed from the Gene Expression Omnibus (GEO) database using accession number GSE162632. This cohort includes 90 male individuals aged between 21 and 69 years. Only 42 PBMCs from European healthy individuals (EUR > 0.9) without any stimulations were included in our study. Selected individuals in this cohort were used in model training and internal testing. Library preparation was done using Single Cell 3′ Reagent Kits v2 User Guide (10x Genomics). PBMCs were sequenced by Illumina NovaSeq 6000. CellRanger (v3.0.2) was used to generate a gene expression matrix. The processed RDS file was downloaded, and raw counts were extracted from the Seurat object.

#### COVID-19-the Netherland cohort

The Data4 dataset^24^ was accessed from European Genome-Phenome Archive (EGA) database using accession number EGAS00001005529. This cohort includes European individuals aged above 17 years. Nine healthy individuals were selected in model training, and 15 convalescence patients were used in the case study. Library preparation was done using Chromium Next GEM Single Cell 3′ Reagent Kits v3.1 (Dual Index), and PBMCs were sequenced by Illumina NovaSeq 6000. CellRanger (v4.0.0) was used to generate a gene expression matrix.

#### BCG cohort

The Data5 dataset^20^ was accessed from the EGA database using accession number EGAS00001006990. This cohort includes 38 European individuals aged above 17 years. To make results comparable, we selected 32 individuals aged between 18 and 30 years. PBMCs without any stimulations were selected in model training. BCG vaccination condition was used in the case study. Library preparation used Chromium Next GEM Single Cell 3′ Library & Gel Bead Kit v3.1. PBMCs were sequenced by Illumina NovaSeq 6000. CellRanger (v3.1.0) was used to generate a gene expression matrix.

#### COVID-19-Germany cohort

The Data6 dataset^55^ was accessed from FASTGenomics (https://beta.fastgenomics.org/p/schulte-schrepping_covid19), which is a cohort including 119 European individuals from Berlin and Bonn; 25 healthy controls did not have detailed age information from the Berlin cohort. In total, 19 healthy individuals and 22 patients with COVID-19 from the Bonn cohort were included in the case study. Individuals’ ages were given as a range within 5 years, and therefore we calculated the mean age as the real chronological age. Library preparation was done using 10x Single Cell 30 Reagent Kit v3.1. PBMCs were sequenced in paired-end mode using Illumina NovaSeq 6000. CellRanger (v3.1.0) was used to generate a gene expression matrix.

#### Thyroid carcinoma cohort

The Data7 dataset^28^ was accessed from the EGA database using accession number EGAS00001005594. This cohort includes 36 individuals aged above 17 years. Eight healthy individuals were used in external validation. Library preparation used 10x Genomics Chromium Single-Cell 3′ v2 RNA-sequencing. PBMCs were sequenced by NextSeq500-v2 150 cycle kit. CellRanger (v3.1.0) was used to generate a gene expression matrix.

#### COVID-19-MHH cohort

The Data8 dataset^56^ was accessed from the EGA database using accession number EGAD00001009331. This cohort includes 78 European individuals aged above 17 years old. Included in the case study were 32 convalescent patients and 46 hospitalized patients with mild or severeCOVID-19. Library preparation used Next GEM Single Cell 30 Reagent Kits v3.1 (Dual Index). PBMCs were sequenced by Illumina NovaSeq 6000. CellRanger (v4.0.0) was used to generate a gene expression matrix.

#### Sepsis cohort

The Data9 dataset^31^ is a cohort including 22 European individuals. Six healthy controls were included in external validation. Library preparation used 10x Genomics Chromium Single Cell 3 v2 RNA sequencing specification. PBMCs were sequenced by NextSeq500-v2 150 cycle kit. CellRanger was used to generate a gene expression matrix.

#### Influenza cohort

The Data10 dataset^29^ was accessed from the EGA database using accession number EGAS00001005446. This cohort includes 10 healthy European individuals aged above 17 years. We only included PBMCs without any stimulations into our study as an external validation dataset. CellRanger (v4.0.0) was used to generate a gene expression matrix. Library preparation used Chromium Next GEM Single Cell 3′ Reagent Kits v3.1 (Dual Index). PBMCs were sequenced by Illumina NovaSeq 6000.

#### MMR cohort

The Data11 dataset^30^ was accessed from the EGA database using accession number EGAS00001006787. This cohort includes European individuals aged above 17 years. Five placebo-vaccinated individuals were used as external validation. Four MMR-vaccinated individuals were used for BCG cohort replication. CellRanger (v4.0.0) was used to generate a gene expression matrix. Library preparation used Chromium Next GEM Single Cell 3′ Library & Gel Bead Kit v3.1 and Chromium Next GEM Chip G Single Cell Kit (10x Genomics). PBMCs were sequenced by Illumina NovaSeq 6000.

#### Gout cohort

The Data12 dataset^32^ is a cohort including European individuals aged above 17 years. Nine healthy individuals were included for external validation. CellRanger (v4.0.0) was used to generate a gene expression matrix. Library preparation used Chromium Next GEM Single Cell 3ʹ Reagent Kits v3.1-Dual Index (CG000315, 10x genomics). PBMCs were sequenced by Illumina NovaSeq 6000.

#### COVID-19-Yale cohort

The Data13 dataset^41^ was accessed from the GEO database using accession number GSE161918. This cohort includes European individuals aged above 17 years. Included in the case study were 33 SARS-CoV-2-infected patients. CellRanger (v3.1.0) was used to generate a gene expression matrix. Library preparation used 10 × 50 Chromium Single Cell Immune Profiling Next GEM v1.1 chemistry. PBMCs were sequenced by Illumina NovaSeq platform.

#### Long-COVID cohort

The Data14 dataset was accessed from the EGA database using accession number EGAS50000000142. This is a single-nuclear dataset, with European individuals aged above 17 years. PBMCs were collected from five time points: acute phase and 3, 6, 9 and 15 months after positive PCR test result. Data were used in the case study. Cell Ranger ARC (v2.0.2) was used to generate a gene expression matrix. Library preparation used 10× Single Cell Multiome ATAC + Gene Expression chemistry. PBMCs were sequenced using Illumina NovaSeq 6000 platform.

### Cell-type-specific transcriptome aging clock

In our study, the model was trained on a large dataset of 1,081 samples, using the chronological age of the samples as the ground truth. The main focus here was to establish cell-type-specific aging clocks for predicting the chronological age of various human immune cells based on scRNA-seq data. This was achieved by selecting marker genes that exhibited age-related expression changes specific to each cell type. The underlying assumption here is that the expression changes of these specific marker genes are strongly correlated with the passage of time and reflect the biological aging process. By identifying specific marker genes, the transcriptome aging clock provides insights into the molecular mechanisms underlying aging. This can help us in understanding the biological pathways driving aging and age-related diseases.

### Preprocessing and analysis of published single-cell datasets

The first five cohorts were used in model training and internal testing. Raw counts from the Seurat object were extracted. Seurat (v4.0)^69^ was used to create the new object. Cells with less than 200 or greater than 5,000 features and with mitochondrial percentage greater than 25% were excluded. Genes expressed in less than 3 cells were also excluded. Thereafter, the data matrix was normalized and scaled by using NormalizeData() and ScaleData() functions. RunPCA() was performed for dimension reduction based on the identified top 2,000 most variable genes before harmony integration^70^. Subsequently, RunNeighbors() and RunCluster() were used to cluster cells based on the shared nearest-neighbor method. In summary, five datasets were integrated and clustered. Annotation was based on manually selected marker genes (B cells: *MS4A1*; CD4^+^ T cells: *IL7R*, *CD3D*; CD8^+^ T cells: *CD8A*, *CD8B*, *CD3D*; monocytes: *CD14*, *FCGR3A*, *LYZ*; NK cells: *GNLY*, *NKG7*; platelets: *PPBP*; dendritic cells: *CD74*) expression levels in each cell type^71^. For data visualization, we used Uniform Manifold Approximation and Projection based on the harmony data integration.

### Feature selection

We first calculated the average expression of each gene in each individual by using the AverageExpression() function. Then we correlated each gene with real age and corrected the *P* value using Benjamini–Hochberg method and filtered out genes with adjusted *P* > 0.05. MIRA^72^ (1.0.4) is a variational auto-encoder-based model to jointly integrate scRNA-seq data and single-cell approaches for the assay for transposase-accessible chromatin using sequencing (scATAC-seq) data. One of the advantages of using this method is it enables us to find the interpretable latent space. We used MIRA’s topic model, which is a generative probabilistic model, as an unsupervised method for feature selection. MIRA found the strongly associated features with the latent topics by calculating the normalized activation of a specific gene given a topic. In addition to Pearson correlation, mutual information^73^ was also used in feature selection. Different from the linear correlation method, mutual information could capture the non-linear relationship. Considering the non-linearity of the scRNA-seq dataset, we adopted the union set of results from Pearson correlation and mutual information. To select the informative gene lists from two methods, we adopted a widely used non-parametric method in biology, rank product^74^. Simply, we permuted the ranked features 100 times and then calculated the rank product of each gene in 100 permuted matrices. Next, we counted the times *c*, in which the rank of the gene is smaller than the observed rank product. We selected those genes with adjusted *E*(rp)/rank < 0.05, where *E*(rp) = *c*/100; rank is the observed rank of the gene.

Specifically, these strategies included (1) examining the correlation between gene expression and chronological age, selecting significant genes based on Benjamini–Hochberg adjusted *P* < 0.05 within each cell type. To determine the optimal number of features to incorporate into the model, we input the ranked features with the step size to 100. Therefore, this approach enabled us to pinpoint the optimal hyperparameters for each specific cell type; (2) applying grid search on the gene set identified in (1) to identify the appropriate number of top features; (3) using the MIRA method for unsupervised feature selection; and (4) using the rank product to select the informative features identified by mutual information and Pearson correlation. All selected methods described below used the same feature selection strategies to ensure comparability between models.

### Converting scRNA-seq into pseudo-cells

Given the sparsity of scRNA sequencing data, we applied a pseudo-cell conversion. We investigated the BootstrapCell method and EnsembleCell method proposed in a previous study^18^ to convert single cells into pseudo-cells, with both methods using LASSO regression for model training. In internal validation, the bootstrapping approach consistently outperformed the ensemble method (Extended Data Table 5). Therefore, we used the BootstrapCell method in our study. In detail, cells from one cell type one individual were down-sampling by 100 times. In each iteration, 15 cells were randomly selected, and the average value of each gene was calculated.

### Model establishment and evaluation criteria

In this study, we selected LASSO regression, random forest, and PointNet to evaluate a range of methodological approaches. LASSO was chosen for its ability to identify linear associations and its stringent feature selection, which is crucial given the high correlation among genes. Random forest was included for its effectiveness in capturing nonlinear relationships and efficient parallel computation. In addition, we explored PointNet as a deep learning method that processes data as sets of points rather than relying on spatial arrangements, making it suitable for scRNA-seq data. Specifically, LASSO regression applies a penalty to the absolute value of the coefficients, effectively driving some coefficients to 0 to reduce overfitting. Furthermore, LASSO shows greater accuracy and robustness compared to ElasticNet (data not shown). For these reasons, we have selected LASSO for our downstream analysis. Fivefold cross-validation was used in the LASSO regression model, using the glmnet^75^ package in R (v4.2.1). To balance the age distribution, we counted the number of individuals at each age ${n}_{i},{i}=18,\ldots ,97$ and calculated the added 1/*n*_*i*_ as weight for each individual. We set 500 trees in the random forest model, using the sklearn.ensemble package in python (v3.9.8). We named these methods as LASSO, MIRA + LASSO, MIRP + LASSO, RF, MIRA + RF, and MIRP + RF. We calculated the median predicted age for each individual measured using Pearson correction, r.m.s.e., and m.a.e. between median predicted age and the actual chronological age to estimate the accuracy of the model. Actual chronological age was defined as years since birth.

### PointNet structure

We investigated a deep learning framework, PointNet^27^, as a prediction model in our study. Specifically designed for tasks involving point cloud data within the realm of deep learning, PointNet is suited for the subset of points with unordered characteristics. Therefore, it could be potentially applied and explored in the scRNA-seq dataset. In particular, the cells can be formulated as ${\{P}_{i}{|i}=\mathrm{1,2},\ldots ,M\}$, where *P*_*i*_ is a vector of the gene expression profile of cell_*i*_. To enable the feature extraction to be more robust and invariant to geometric transformations, we introduced the feature transform T-Net in our study. The structure of T-Net included three 1D convolutional layers (the numbers of filters are 32, 64, 256) with kernel size of 1 along the cell, followed by a maxpooling layer and three fully connected layers. In our PointNet model, we initially used four 1D convolutional layers, with the number of filters being 128, 64, 32, and 32. Subsequently, T-Net was used and connected by a 1D convolutional layer with 32 filters. To jointly reason the low-level features and high-level features, we further incorporated the architecture of U-Net in layers 5 through 7, which were skip-connected to the 3rd, 2nd, and 1st layers, respectively. Finally, three fully connected layers were designed to progressively aggregate the features and regress the label of cells. Except the last layer using linear activation, in each layer, BatchNormalization and rectified linear unit (ReLU) activation were used.

### Description of established cell-type-specific aging clocks

Genes with nonzero coefficients were selected for downstream analysis. Genes with positive coefficients represented the higher expression level in the elderly, and vice versa. To check the specificity of the aging clock tailed for each cell type, we used the UpSet() function^76^ to show the intersection and uniqueness. To clarify the contributing marker genes were specific in each cell type, we ranked the marker genes based on the absolute value of their coefficients and calculated the trajectory of marker genes over age in each cell type. We calculated the average expression level per gene per age by using the AverageExpression() function in Seurat and visualized it by ggplot2().

### TAA analysis in vaccination or disease cohorts

Considering the individualized differences, for the BCG cohort, we only selected the individuals who were participants in both before and after vaccination sampling. Aging alterations induced by vaccination were compared within each individual between two time points. For infectious diseases, to keep the predicted age comparable, we selected patients and healthy controls having comparable actual chronological ages and performed the same analysis as mentioned above. For COVID-19 cohorts, four groups of individuals with real chronological ages of 38, 31, and 39 years (for healthy, mild, and severe), as well as 57, 52, and 52 years (for corresponding conditions), in addition to 63 years (representing all conditions) and 69 years (all groups) were selected. To show the aging alterations after infection, we additionally adopted the strategy proposed by ref. ^77^ to adjust the real actual age based on our model. In brief, we first fit a linear regression between the predicted bias and actual chronological age. Then the actual chronological age was adjusted by the fitted model.

### Differentially expressed genes identification

In the 300BCG cohort, scRNA-seq data were collected at before (*T*_*0*_) and three months after (*T*_*3m*_) BCG vaccination. Differentially expressed genes (DEGs) were calculated in the following comparisons: (1) AR versus AA at both *T*_0_ and *T*_3m_, respectively, and (2) the comparison between *T*_3m_ and *T*_0_ within AR and AA, respectively. FindMarkers() function was used, and significant genes (adjusted *P* < 0.05, two-sided) were considered for downstream analysis.

### The assessment of effects of marker genes in vaccination or infectious diseases cohorts

For the vaccination cohort, we calculated the fold change of the marker genes by using FindMarkers() function and selected those with *P* value below 0.05 (two-sided test) for downstream analysis. For the infectious disease cohort, genes were selected from the intersections between marker genes and DEGs.

### Pathway enrichment analysis

We used the online database of FUMA^78^ (https://fuma.ctglab.nl) for enrichment analysis. The databases used for enrichment analysis are the following: WikiPathways (v20191010), MsigDB (v2023.1.Hs), Kyoto Encyclopedia of Genes and Genomes (KEGG) (MsigDB c2), Reactome (MsigDB c2), Gene Ontology (GO) biological processes (MsigDB c5), and GO molecular functions (MsigDB c5). Only pathways with Benjamini–Hochberg adjusted *P* < 0.05 (two-sided test) were considered. ggplot2 package in R (v3.4.1) was used to visualize the enrichment results.

### IFN response genes score in the MMR cohort

We applied our aging clocks to a MMR vaccine cohort^30^, before vaccination and 4 weeks after vaccination. Four MMR-vaccinated individuals and five placebo-vaccinated individuals aged below 30 years were included in this study. We calculated the differential expression level of IFN response genes collected from the hallmark gene set^79^. We used those genes with *P* < 0.05 (two-sided test) and calculated the gene set score for MMR-vaccinated and placebo-vaccinated groups, respectively.

### Transcription factors identification in the BCG cohort

DEGs identified from comparisons between before and after vaccination in AA group or AR group were set as input to the R package: RcisTarget (v1.16.0)^50^. We used hg38__refseq-r80__10kb_up_and_down_tss.mc9nr.feather as the reference database. We used those transcription factors with “directAnnotation” for downstream analysis. The R package GENIE3 (v1.18.0)^80^ was adopted to reconstruct the gene regulatory network for the AA group and AR group, respectively. Based on the importance score, only the top 100 transcription factor–target links were considered, and we compared the links before and after vaccination within AA and AR groups, respectively. Only unique links that lead to age alterations were considered. Cytoscape v3.9.1^81^ was used for transcription factor–target network visualization.

### Statistics and reproducibility

No statistical methods were used to predetermine sample sizes, but our sample sizes are similar to or larger than those reported in previous publications. We excluded individuals without age information, those aged below 18 years, and individuals not of European ancestry. The training set and internal validation set were randomly selected, and this analysis was repeated 10 times. Data distribution was assumed to be normal, but this was not formally tested. Data collection and analysis were not performed blind to the conditions of the experiments.

### Reporting summary

Further information on research design is available in the Nature Portfolio Reporting Summary linked to this article.

## Supplementary information

Reporting Summary

Supplementary Table 1 Model features and coefficients.

## Source data

Source Data Fig. 2 Statistical source data.

Source Data Fig. 3 Statistical source data.

Source Data Fig. 4 Statistical source data.

Source Data Fig. 5 Statistical source data.

Source Data Extended Data Fig. 2 Statistical source data.

Source Data Extended Data Fig. 3 Statistical source data.

Source Data Extended Data Fig. 4 Statistical source data.

## Figures and Tables

**Extended Data Table 1:**
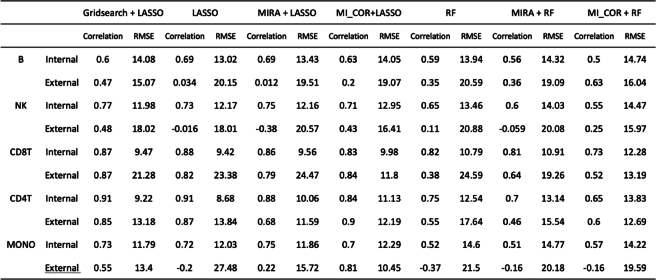
Methods comparison

**Extended Data Table 2:**
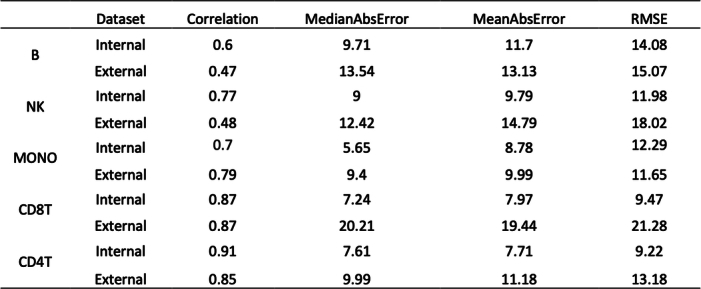
Internal and external validation performance

**Extended Data Table 3:**
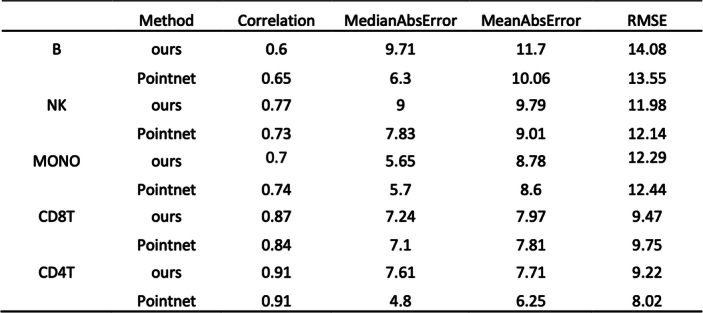
PointNet evaluation

**Extended Data Table 4:**
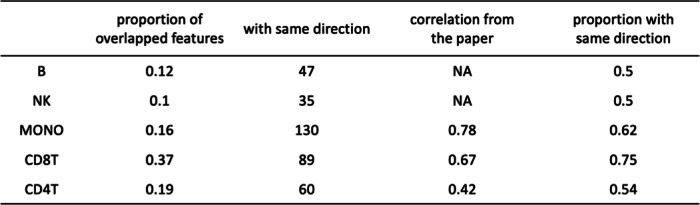
Replication with public data

**Extended Data Table 5:**
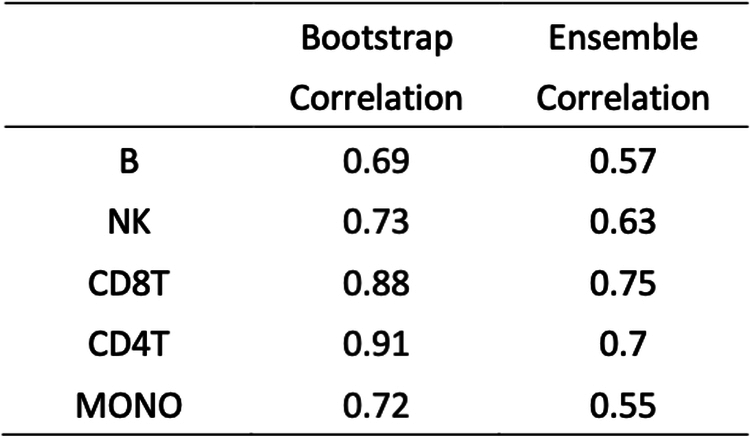
PseudoCell Method evaluation

## Data Availability

OneK1K cohort data were obtained from CELLxGENE (https://cellxgene.cziscience.com/collections/dde06e0f-ab3b-46be-96a2-a8082383c4a1). COVID-19-UK cohort data were obtained from European Molecular Biology Laboratory's European Bioinformatics Institute (EMBL-EBI) (https://www.ebi.ac.uk/biostudies/arrayexpress/studies/E-MTAB-10026). Influenza A virus (IAV) cohort data were obtained from Gene Expression Omnibus (GEO) (GSE162632). COVID-19-the Netherland cohort data were obtained from the European Genome-Phenome Archive (EGA) (EGAS00001005529). BCG cohort data were obtained from EGA (EGAS00001006990). COVID-19-Germany cohort data were obtained from FASTGenomics (https://beta.fastgenomics.org/p/schulte-schrepping_covid19). Thyroid carcinoma cohort data were obtained from EGA (EGAS00001005594). COVID-19-MHH cohort data were obtained from EGA (EGAD00001009331). Sepsis cohort data were obtained from ref. ^31^. Influenza cohort data were obtained from EGA (EGAS00001005446). MMR cohort data were obtained from EGA (EGAS00001006787). Gout cohort data were obtained from ref. ^32^. COVID-19-Yale cohort data were obtained from GEO (GSE161918). Long-COVID cohort data were obtained from EGA (EGAS50000000142). The databases used for enrichment analysis are the following: WikiPathways (v20191010), MsigDB (v2023.1.Hs), Kyoto Encyclopedia of Genes and Genomes (KEGG) (MsigDB c2), Reactome (MsigDB c2), Gene Ontology (GO) biological processes (MsigDB c5), and GO molecular functions (MsigDB c5).

## Code availability

The codebase and R package for the established aging clocks, sc-ImmuAging, is available via GitHub at https://github.com/CiiM-Bioinformatics-group/scImmuAging.
