# Supplementary material for: Single-cell immune aging clocks reveal inter-individual heterogeneity during infection and vaccination
Source: Nat Aging. 2025 Mar 5;5(4):607–21. doi: 10.1038/s43587-025-00819-z (PMC12003178; doi:10.1038/s43587-025-00819-z)
Supplement: Supplementary file 1 — Reporting Summary [file 43587_2025_819_MOESM1_ESM.pdf]

nature portfolio

Corresponding author(s): Yang Li

Last updated by author(s): Jan 8, 2025

Reporting Summary

Nature Portfolio wishes to improve the reproducibility of the work that we publish. This form provides structure for consistency and transparency in reporting. For further information on Nature Portfolio policies, see our Editorial Policies and the Editorial Policy Checklist.

Statistics

For all statistical analyses, confirm that the following items are present in the figure legend, table legend, main text, or Methods section.

n/a	Confirmed	
☐	☒	The exact sample size (n) for each experimental group/condition, given as a discrete number and unit of measurement
☐	☒	A statement on whether measurements were taken from distinct samples or whether the same sample was measured repeatedly
☐	☒	The statistical test(s) used AND whether they are one- or two-sided<br>*Only common tests should be described solely by name; describe more complex techniques in the Methods section.*
☒	☐	A description of all covariates tested
☒	☐	A description of any assumptions or corrections, such as tests of normality and adjustment for multiple comparisons
☐	☒	A full description of the statistical parameters including central tendency (e.g. means) or other basic estimates (e.g. regression coefficient) AND variation (e.g. standard deviation) or associated estimates of uncertainty (e.g. confidence intervals)
☐	☒	For null hypothesis testing, the test statistic (e.g. F, t, r) with confidence intervals, effect sizes, degrees of freedom and P value noted<br>*Give P values as exact values whenever suitable.*
☒	☐	For Bayesian analysis, information on the choice of priors and Markov chain Monte Carlo settings
☒	☐	For hierarchical and complex designs, identification of the appropriate level for tests and full reporting of outcomes
☐	☒	Estimates of effect sizes (e.g. Cohen's d, Pearson's r), indicating how they were calculated

Our web collection on statistics for biologists contains articles on many of the points above.

Software and code

Policy information about availability of computer code

Data collection	R 4.2.1, python 3.9.8
Data analysis	R packages: Seurat 4.0, FUMA 1.5.2, ggplot2 3.4.1, readr 2.1.3, tidyverse 1.3.2, glmnet 4.1.4, ggpubr 0.4.0, biomaRt 2.52.0, infotheo 1.2.0.1, purrr 0.3.4, ggridges 0.5.4, ComplexHeatmap 2.12.1, RcisTarget 1.16.0, GENIE3 1.18.0; python packages: pandas 1.4.4, numpy 1.19.5, tensorflow 2.5.3, keras 2.5.0, scipy 1.9.3, sklearn 0.23.2, scanpy 1.9.13, mira 1.0.4, Biorender<br>code: https://github.com/CiiM-Bioinformatics-group/scImmuAging)

For manuscripts utilizing custom algorithms or software that are central to the research but not yet described in published literature, software must be made available to editors and reviewers. We strongly encourage code deposition in a community repository (e.g. GitHub). See the Nature Portfolio guidelines for submitting code & software for further information.

Data

Policy information about availability of data

All manuscripts must include a data availability statement. This statement should provide the following information, where applicable:

- Accession codes, unique identifiers, or web links for publicly available datasets
- A description of any restrictions on data availability
- For clinical datasets or third party data, please ensure that the statement adheres to our policy

OneK1K cohort data from CELLxGENE (https://cellxgene.cziscience.com/collections/dde06e0f-ab3b-46be-96a2-a8082383c4a1), COVID-19-UK cohort data from

EMBL-EBI (https://www.ebi.ac.uk/biostudies/arrayexpress/studies/E-MTAB-10026), IAV cohort data from Gene Expression Omnibus (GEO, GSE162632), COVID-19-the Netherland cohort data from European Genome-Phenome Archive (EGA, EGAS00001005529), BCG cohort data from EGA (EGAS00001006990), COVID-19-Germany cohort data from FASTGenomics (https://beta.fastgenomics.org/p/schulte-schrepping_covid19), Thyroid Carcinoma cohort data from EGA (EGAS00001005594), COVID-19-MHH cohort data from EGA (EGAD00001009331), Sepsis cohort data from Grondman et al.31, Influenza cohort data from EGA (EGAS00001005446), MMR cohort data from EGA (EGAS00001006787), Gout cohort data from Alaswad et al.32, COVID-19-Yale cohort data from GEO (GSE161918), Long COVID cohort data from EGA (EGAS50000000142). Database for enrichment analysis: WikiPathways (v20191010), MsigDB (v2023.1.Hs), KEGG (MsigDB c2), Reactome (MsigDB c2), GO biological processes (MsigDB c5), GO molecular functions (MsigDB c5).

Research involving human participants, their data, or biological material

Policy information about studies with human participants or human data. See also policy information about sex, gender (identity/presentation), and sexual orientation and race, ethnicity and racism.

Reporting on sex and gender	N/A
Reporting on race, ethnicity, or other socially relevant groupings	N/A
Population characteristics	N/A
Recruitment	N/A
Ethics oversight	N/A

Note that full information on the approval of the study protocol must also be provided in the manuscript.

Field-specific reporting

Please select the one below that is the best fit for your research. If you are not sure, read the appropriate sections before making your selection.

☒ Life sciences ☐ Behavioural & social sciences ☐ Ecological, evolutionary & environmental sciences

For a reference copy of the document with all sections, see nature.com/documents/nr-reporting-summary-flat.pdf

Life sciences study design

All studies must disclose on these points even when the disclosure is negative.

Sample size	The cell type-specific aging clocks for human PBMC are established based on 1.3 million cells from scRNA-seq datasets. We only included individuals aged above 18 years old with European ancestry. And the data should provide detailed age information. In total, our study comprises 1,081 individuals. We randomly split them into training set (80%) and internal testing set (20%). Additionally, we also established an independent external dataset including 42 individuals to further validate the accuracy of our model. To reduce bias, we only focus on main cell types (monocytes, NK cells, B cells, CD8+ T cells and CD4+ T cells) with sufficient cell counts in PBMC, ensuring robust results for each cell type. To our knowledge, the sample size of our study is comparable to or exceeds that of other aging clock studies. Therefore, this dataset is sufficient to provide reliable and accurate results.
Data exclusions	We included all the healthy individuals aged above 18 years old for model training and internal/external testing. COVID-19 patients or vaccinated individuals are used as case study.
Replication	No new data was generated for this study. The code is available on GitHub: https://github.com/CiiM-Bioinformatics-group/scImmuAging.
Randomization	The training and internal validation samples are randomly assigned. The external dataset is independent with training/internal testing dataset.
Blinding	All datasets used in this study were publicly available. The training data were collected from five independent studies, and the external data were obtained from another five independent studies. The training set and internal validation were randomly assigned. Additionally, we repeated this analysis 10 times. Data collection and analysis were not performed blind to the conditions of the experiments.

Reporting for specific materials, systems and methods

We require information from authors about some types of materials, experimental systems and methods used in many studies. Here, indicate whether each material, system or method listed is relevant to your study. If you are not sure if a list item applies to your research, read the appropriate section before selecting a response.

Materials & experimental systems

n/a	Involved in the study
☒	☐ Antibodies
☒	☐ Eukaryotic cell lines
☒	☐ Palaeontology and archaeology
☒	☐ Animals and other organisms
☒	☐ Clinical data
☒	☐ Dual use research of concern
☒	☐ Plants

Methods

n/a	Involved in the study
☒	☐ ChIP-seq
☒	☐ Flow cytometry
☒	☐ MRI-based neuroimaging

Plants

Seed stocks

Report on the source of all seed stocks or other plant material used. If applicable, state the seed stock centre and catalogue number. If plant specimens were collected from the field, describe the collection location, date and sampling procedures.

Novel plant genotypes

Describe the methods by which all novel plant genotypes were produced. This includes those generated by transgenic approaches, gene editing, chemical/radiation-based mutagenesis and hybridization. For transgenic lines, describe the transformation method, the number of independent lines analyzed and the generation upon which experiments were performed. For gene-edited lines, describe the editor used, the endogenous sequence targeted for editing, the targeting guide RNA sequence (if applicable) and how the editor was applied.

Authentication

Describe any authentication procedures for each seed stock used or novel genotype generated. Describe any experiments used to assess the effect of a mutation and, where applicable, how potential secondary effects (e.g. second site T-DNA insertions, mosiacism, off-target gene editing) were examined.
